# An Innovative Approach to Target Lesion Progression in the Response Evaluation Criteria in Solid Tumors (RECIST) 1.1: The Enaworu 25 mm Nadir Rule

**DOI:** 10.7759/cureus.82715

**Published:** 2025-04-21

**Authors:** Oghenetanure R Enaworu

**Affiliations:** 1 Chao Family Comprehensive Cancer Center, University of California, Irvine, Irvine, USA

**Keywords:** clinical trials, lesion assessment, nadir, oncology, recist 1.1, target lesion progression

## Abstract

The Response Evaluation Criteria in Solid Tumors (RECIST) 1.1 remains the benchmark for evaluating tumour response within clinical trials and oncological practice. It defines disease progression in target lesions as a ≥20% increase in the sum of the longest diameters (SLD), accompanied by an additional absolute increase of ≥5 mm from the nadir. While these dual criteria enhance diagnostic accuracy, their simultaneous application poses several challenges, including the complexity of lesion tracking and potential ambiguity in assessing progression for smaller target lesions. This study presents Enaworu’s 25 mm nadir rule, a novel and standardised approach designed to streamline the assessment of target lesion progression. According to this rule, lesions with a nadir of less than 25 mm progress based solely on an absolute increase of 5 mm, whereas lesions with a nadir of 25 mm or more will progress based solely on a 20% increase from the nadir, thereby eliminating the necessity of applying both criteria concurrently. A simulated dataset comprising 1,000 patients, with nadir tumour sizes varying from 0.03 mm to 69.96 mm, was employed to compare progression classification under RECIST 1.1 and the 25 mm nadir rule. The results indicated identical classification outcomes, with 255 patients (25.5%) satisfying the progression criteria under both methodologies. The simplified rule maintained diagnostic accuracy while alleviating administrative burdens and enhancing clarity. The Enaworu’s 25 mm nadir rule constitutes a practical refinement of RECIST 1.1, optimising workflow efficiency, reducing ambiguity, and ensuring consistency within clinical and research settings.

## Introduction

The Response Evaluation Criteria in Solid Tumors (RECIST) 1.1 [1] provides a standardised framework for assessing tumour response, a key component in clinical trials and routine oncologic practice. Under RECIST 1.1, disease progression in target lesions is defined by a ≥20% increase in the sum of the longest diameters (SLD) of target lesions, with an additional absolute increase of ≥5 mm from the nadir (smallest recorded SLD) [2]. The inclusion of the 5 mm absolute increase criterion in RECIST 1.1 was to prevent the premature classification of progressive disease (PD) in cases where the baseline tumour burden is small [3]. While these dual criteria improve diagnostic accuracy, their concurrent application presents practical challenges, requiring meticulous tracking and precise calculations that may hinder workflow efficiency. A study analysing data from over 50 clinical trials across multiple malignancies highlighted this issue as a key challenge in RECIST 1.1 [4]. Clinicians and researchers must assess a 20% increase and an absolute increase of at least 5 mm to determine progression. This can introduce ambiguity, particularly when small target lesions do not meet the 5 mm threshold despite a 20% increase. This raises an important question: At what point should a lesion be considered “small” in this context?

This study proposes Enaworu’s 25 mm nadir rule to address these challenges, which provides a standardised approach to optimising target lesion progression assessment. Under this rule, for lesions with a nadir <25 mm, the progression threshold will be defined solely by an absolute increase of 5 mm, while for lesions with a nadir ≥25 mm, only the 20% increase criterion will be applied. For example, for lesions with a nadir of 24 mm and 30 mm, the progression thresholds will be set at 29 mm and 36 mm, respectively. This modification eliminates the need to apply both criteria, streamlining the assessment process while preserving diagnostic accuracy and reducing ambiguity.

Previous studies [5-8] have refined RECIST 1.1, clarified its limitations, and explored challenges in data extraction and interpretation in clinical trials. Other studies [9-13] have proposed theoretical frameworks and alternative progression methods; however, none have conceptualised a threshold for target lesion progression based on either a 20% increase or a 5 mm absolute increase, rather than requiring both. The 25 mm nadir rule addresses this gap by establishing a standardised threshold that simplifies progression assessment while maintaining diagnostic accuracy.

This study aims to compare the assessments of target lesion progression between the proposed Enaworu’s 25 mm nadir rule and the traditional RECIST 1.1 to determine whether both methods yield comparable outcomes.

## Materials and methods

Mathematical model equation and rationale of the 25 mm threshold

To identify the critical point at which the two progression criteria of RECIST 1.1 converge, we derived the following equation:

$1.2 \times N = N + 5 \text{mm}$

$1.2 \times N - N = 5 \text{mm}$

$(1.2 - 1) \times N = 5 \text{mm}$

$0.2 \times N = 5 \text{mm}$

$N = \frac{5 \text{mm}}{0.2}$

$N = 25 \text{mm}$

In this equation, “1.2 × *N*” denotes the calculation for a 20% rise in tumour size (with *N* representing the nadir in mm). The expression “*N* + 5” indicates the required 5 mm increase from the nadir, as the RECIST 1.1 criteria stipulate to signify progression. When *N* equals 25 mm, the criteria for a 20% increase and the absolute 5 mm increase align. A nadir of 25 mm marks the key point where a 20% rise corresponds to a 5 mm absolute increase, satisfying both the percentage and absolute progression standards of RECIST 1.1.

Study design and population

This study utilised a simulated dataset to evaluate target lesion progression based on the RECIST 1.1 criteria and the 25 mm nadir rule. The dataset was generated to represent a cohort of 1,000 patients, with the distribution of baseline nadir tumour sizes (measured in millimetres) ranging from 0.03 to 69.96. To assess the impact of the critical threshold, the simulated dataset included nadir values of 24.9 mm and 25 mm, highlighting a potential shift in progression criteria based on tumour size.

Progression Criteria

Two distinct criteria for tumour progression were employed in the dataset analysis. Firstly, the RECIST 1.1 criteria stipulate that tumour progression is defined as an increase of 20% or more from the nadir measurement, accompanied by a minimum absolute increase of 5 mm. Secondly, the 25 mm nadir rule was implemented, whereby tumours with a nadir size of less than 25 mm are deemed progressive if they demonstrate an absolute increment of at least 5 mm. In contrast, tumours with a nadir size of 25 mm or more significant are classified as progressive based on a 20% or greater increase in size. For this study, the outcomes were categorised into progressive and non-progressive diseases.

Data Generation and Statistical Analysis

Tumour sizes were generated randomly for each patient, utilising a uniform distribution. Subsequently, follow-up tumour sizes were simulated based on a percentage increase, with the follow-up size determined as a function of the baseline nadir and a random increase ranging from 0% to 30%. To comprehensively evaluate tumour progression, the RECIST 1.1 and the 25 mm nadir rule were systematically applied across the entire cohort. A visual comparison of both progression criteria was presented through side-by-side scatter plots. Furthermore, an enlarged visualisation centred on the critical nadir size range (20 mm to 30 mm) was provided, particularly underscoring tumours with 24.9 mm and 25 mm measurements, where the divergence between the two criteria is most significant. Data generation and statistical analyses were conducted utilising R version 4.2.3 (R Foundation for Statistical Computing, Vienna, Austria).

## Results

The distribution of baseline tumour sizes revealed a minimum value of 0.03 mm, a first quartile (Q1) of 17.75 mm, a median of 34.30 mm, a mean of 34.81 mm, a third quartile (Q3) of 52.28 mm, and a maximum value of 69.96 mm. These figures illustrate a wide range of tumour sizes, with the majority falling between 17.75 mm and 52.28 mm. The follow-up distribution of tumour sizes was similarly structured.

Tumour progression analysis

Table 1 presents a comparative analysis of tumour progression classification under RECIST 1.1 and the 25 mm nadir rule, summarising the percentage of patients meeting the progression criteria for each method.

**Table 1:** Comparison of tumour progression classification between RECIST 1.1 and Enaworu's 25 mm nadir rule. RECIST: Response Evaluation Criteria in Solid Tumors.

Progression status	RECIST 1.1 (n, %)	Enaworu’s 25 mm nadir rule (n, %)
Progression (True)	255 (25.5%)	255 (25.5%)
No progression (False)	745 (74.5%)	745 (74.5%)

Analysis revealed that using the RECIST 1.1 progression criteria for target lesion, 255 patients (25.5%) showed progressive disease, while 745 patients (74.5%) were non-progressive. Conversely, the 25 mm nadir rule showed similar results, demonstrating complete concordance with RECIST 1.1.

Critical Tumour Size Threshold (24.9 mm and 25 mm)

Table 2 below shows the progression classifications for both methods at these specific tumour sizes. RECIST 1.1 and the 25 mm nadir rule classified tumours identically at these critical thresholds, reinforcing their overall concordance in progression assessment.

**Table 2:** Tumour progression classification at critical nadir thresholds. RECIST: Response Evaluation Criteria in Solid Tumors.

Nadir size (mm)	RECIST 1.1, progression (n, %)	RECIST 1.1, no progression (n, %)	Enaworu's 25 mm nadir rule, progression (n, %)	Enaworu's 25 mm nadir rule, no progression (n, %)
24.9	50 (50%)	50 (50%)	50 (50%)	50 (50%)
25	70 (70%)	30 (30%)	70 (70%)	30 (30%)

**Figure 1:**
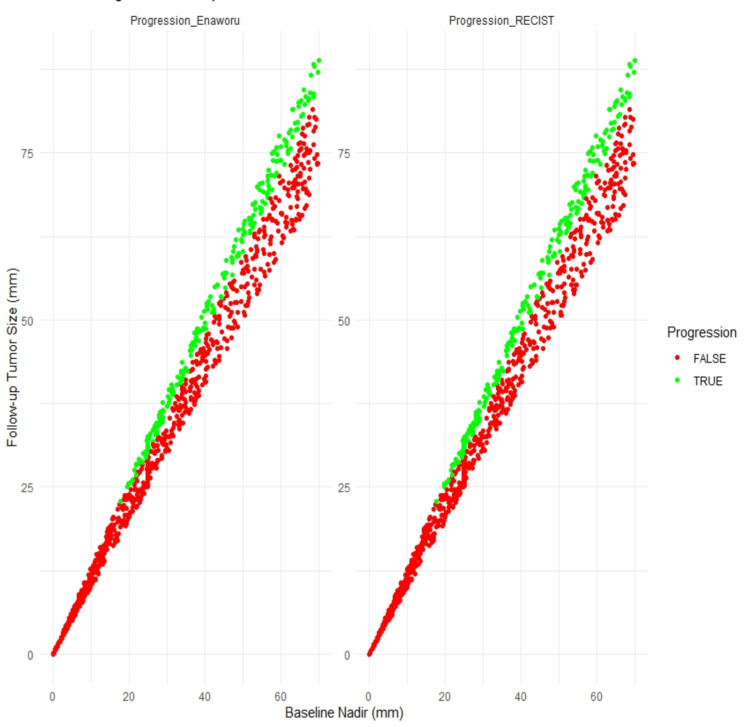
Comparison of tumour progression between RECIST 1.1 and Enaworu's 25 mm threshold rule, illustrating changes in tumour size and progression status. RECIST: Response Evaluation Criteria in Solid Tumors.

**Figure 2:**
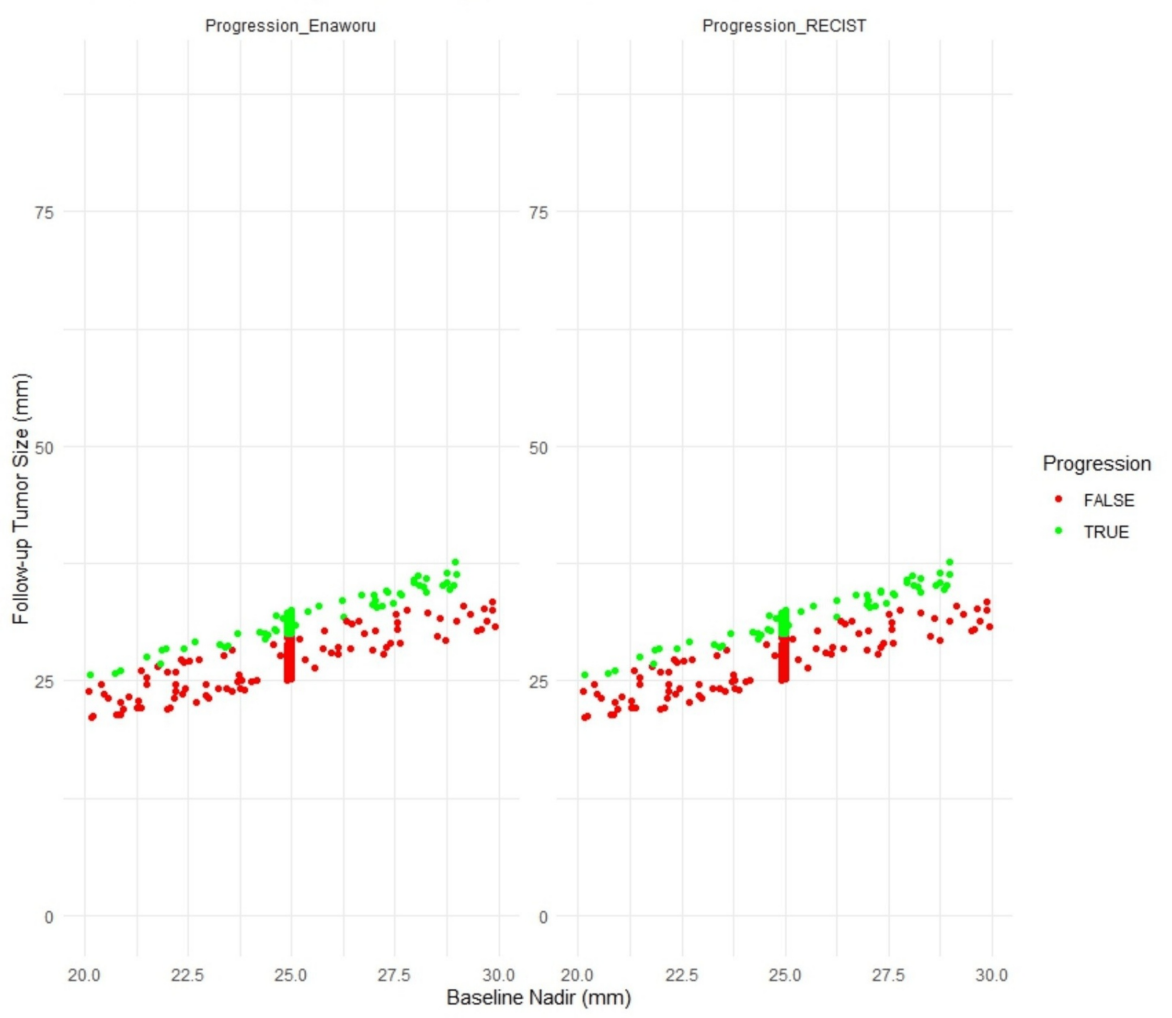
Comparison of tumour progression around the critical values of 24.9 mm and 25 mm. RECIST: Response Evaluation Criteria in Solid Tumors.

Figure 1 displays both methods’ side-by-side scatter plots comparing baseline tumour size (nadir) with follow-up tumour size (in mm). Each data point represents a patient and is colour-coded to indicate progression status (red for progression, green for no progression). This visualisation highlights the concordance between the two methods in classifying target lesion progression. Figure 2 emphasises the critical tumour size range of 20 mm to 30 mm, further demonstrating the alignment between the two methods.

## Discussion

This study showed that the 25 mm nadir rule aligns with RECIST 1.1 in classifying target lesion progression. Both methods identified 25.5% of patients with progression and 74.5% with non-progression, demonstrating complete concordance. This suggests that the 25 mm nadir rule can replicate the progression classification provided by RECIST 1.1 while offering a simplified approach to tumour assessment, particularly for small lesions. The findings support the accuracy and reliability of RECIST 1.1, as it has been shown to provide consistent and reproducible results in response assessment [14].

The Enaworu’s 25 mm nadir rule stipulates that, under RECIST 1.1 criteria, the progression threshold for target lesions exhibiting a nadir measurement of less than 25 mm is solely determined by an absolute increase of 5 mm. Conversely, for target lesions with a 25 mm or greater nadir measurement, the progression threshold is defined by a 20% increase from the nadir. The rationale is based on the observation that if the nadir measurement is less than 25 mm, a 20% increase yields a value below 5 mm, failing to meet the RECIST 1.1 criteria for target lesion progression. However, a 5 mm increase is more practical because it automatically satisfies the 5 mm minimum and the desired 20% increase. In contrast, for target lesions with a nadir of 25 mm or more, a 20% increase will invariably meet or exceed the 5 mm threshold, rendering the 5 mm rule redundant.

The findings of this study align with existing literature advocating for simplified tumour progression assessment methods, particularly in the context of personalised tumour response evaluation [15]. By offering a clear, threshold-based solution, the 25 mm nadir rule eliminates the ambiguity of applying the dual criteria of RECIST 1.1, thereby enhancing workflow efficiency. This approach improves efficiency, similar to a study that showed significant reductions in nonconformities and time savings in radiology workflows [16]. While the study [16] focused on optimising radiological processes, the 25 mm nadir rule simplifies progression assessment, reducing the complexity of RECIST 1.1 criteria, ultimately enhancing both efficiency and accuracy.

The 25 mm nadir rule can be included in RECIST 1.1 worksheets used in clinical and trial environments to define a progressive disease (PD) threshold. This adjustment minimises the chances of errors, creating a more intuitive and clinically relevant framework for progression assessment. Additionally, integrating the 25 mm nadir rule into AI-driven RECIST calculators marks a significant step in simplifying tumour progression evaluations in clinical oncology [17]. These tools can automate the rule’s application, effectively managing extensive datasets of tumour measurements and equipping clinicians with prompt, dependable, and uniform outcomes.

However, like any novel approach, Enaworu’s proposed 25 mm nadir rule has limitations. The study relied on simulated data. Future research using real-world clinical datasets would be beneficial for validating the rule’s applicability across a broader range of target lesions and patient populations [18].

## Conclusions

The 25 mm nadir rule presents a practical and simplified approach to tumour progression assessment, addressing key challenges in the RECIST 1.1 criteria. By establishing a single threshold for progression, it eliminates the complexities of using both absolute and relative measures simultaneously. Our findings demonstrate that this method aligns with RECIST 1.1 regarding progression outcomes. This approach can potentially improve clinical decision-making, particularly in routine practice and clinical trials, by streamlining response evaluations. Further validation in diverse, real-world settings is essential to fully assess its impact and potential integration into clinical workflows.
